# Participation patterns of children with cerebral palsy: A caregiver’s perspective

**DOI:** 10.4102/ajod.v12i0.1058

**Published:** 2023-01-31

**Authors:** Lethabo E. Africa, Anri Human, Muziwakhe D. Tshabalala

**Affiliations:** 1 Department of Physiotherapy, Faculty of Healthcare Sciences, Sefako Makgatho Healthcare Sciences University, Pretoria, South Africa; 2 Department of Physiotherapy, Faculty of Health Sciences, University of Pretoria, Pretoria, South Africa

**Keywords:** participation, cerebral palsy, education, leisure, play, children, caregiver

## Abstract

**Background:**

Participation in activities of daily living (ADL), education, leisure and play in children living with cerebral palsy (CP) may be affected by various factors, as outlined in the International Classification of Functioning, Disability and Health Framework (ICF). The aim of this study was to describe the participation patterns of a group of these children.

**Objectives:**

This study aimed to describe participation patterns in ADL, education, leisure and play activities of children living with CP in Modimolle.

**Method:**

An exploratory-descriptive qualitative (EDQ) study design was used. A researcher-constructed bio-demographic data sheet and a semi-structured interview schedule were used to collect data from the primary caregivers of children (5–17 years) living with CP in Modimolle. Interviews were transcribed verbatim, translated from Sepedi to English and analysed using the content analysis approach and NVivo software.

**Results:**

The findings of this study indicated that children living with CP in Modimolle require set-up and assistance to participate in various ADL such as self-care, family and community activities. They also participate in formal and informal educational programmes as well as active and passive leisure and play activities. However, at the moment, they have limited opportunities to participate because of resource constraints and inaccessible infrastructure.

**Conclusion:**

Although children with CP in Modimolle perform some ADL, and participate in educational, leisure and play activities, they are not fully integrated into their community. Legislative support and policy implementation are required to improve participation and integration of children living with CP. Further studies on community-specific integrative strategies to enhance participation among children living with disabilities are recommended.

**Contribution:**

This paper provides valuable information on the participation patterns of children with CP living in a rural area of South Africa. The findings can assist with development and implementation of community-specific, integrative health and social care strategies to enhance participation among children living with disabilities.

## Introduction

Cerebral palsy (CP) is a movement and posture disorder caused by impairment of the developing brain of an infant. This disorder is associated with various combinations of comorbidities such as epilepsy, visual, motor and cognitive impairments (Donald et al. 2014; Reddy 2005; Richards & Malouin 2013). The global prevalence of CP has been reported as 2–2.5 per 1000 births (Donald et al. 2014; Eunson 2012, 2016; Odding, Roebroek & Stam 2006). In Northern Africa, the prevalence is two per 1000 live births, while the prevalence of CP in Southern Africa, including South Africa (SA), is higher, at approximately 10 per 1000 live births (Couper 2002; Donald et al. 2014).

The higher risk and incidence of CP in SA may be attributable to the prevalence of human immunodeficiency virus (HIV) and acquired immunodeficiency syndrome (AIDS), malaria, poverty-related malnutrition, tuberculosis (TB) and meningitis observed in some provinces, such as Limpopo (Treasury.gov.za 2019). Children living with HIV and AIDS may present with neurological impairments caused by the virus, resulting in complications, such as HIV encephalopathy (HIVE) and HIV-associated neurocognitive disorders (HAND) causing injury to the developing brain (Croucher & Winston 2013; Levin 2006; Sørensen Kristian et al. 2016).

Cerebral palsy, with or without comorbidities, may subsequently lead to impairments in the body structure and function domain (International Classification of Functioning, Disability and Health framework [ICF], World Health Organization [WHO] 2001), activity limitations and participation restrictions in education, leisure and play, with a subsequent negative impact on health-related quality of life (HRQoL) of children and their families (Bearden et al. 2016; Donald et al. 2014; Law et al. 2014; Reddy 2005; WHO 2001).

Through participation in education, leisure and play activities, children are provided with opportunities to form friendships, to gain knowledge, to learn new skills and to be creative. This can assist children in finding meaning in life, have a successful transition into adulthood and could foster independence and successful integration into society (Majnemer et al. 2010; Orlin et al. 2010; Palisano et al. 2009; Wagner et al. 2005).

Children living with CP, like all children, desire to learn new skills and, if possible, to engage in meaningful occupations and contribute to their communities (McConachie et al. 2006). Participation in educational, leisure and play activities is essential for a child’s physical, psychological and cognitive development. Research has shown that if given the opportunity, children with disabilities can attain these goals and that participation may have a positive effect on their HRQoL (Bourke-Taylor et al. 2017; Bult et al. 2011; Majnemer et al. 2010; Orlin et al. 2010). The evidence is seen mostly in well-resourced, developed countries, where policies and legislation are implemented to enable children with disabilities to overcome barriers and participate in education, leisure and play activities in their communities (Aron & Loprest 2012; Bourke-Taylor et al. 2017; Power et al. 2018; Radsel, Osredkar & Neubauer 2017; Shikako-Thomas et al. 2013).

There is a paucity of evidence on the participation patterns of children with CP and their engagement in ADL, education, leisure and play activities, especially in low- and middle-income countries such as SA (Abdel Malek, Rosenbaum & Gorter 2020). Children living with disabilities in rural settlements are often at a disadvantage because of limited infrastructure and resources (Maphumulo & Bhengu 2019; Treasury.gov.za 2019). Information on the participation patterns of children with disabilities is required for the development of context-specific policies and legislation. The aim of this study was therefore to explore and describe the participation patterns of children living with CP in a rural area of SA (Modimolle, Limpopo), from the perspective of their caregivers.

## Research methods and design

### Study design

An exploratory descriptive qualitative (EDQ) research approach, as described by Hunter, McCallum and Howes (2019), was used. Data were collected through direct interactions with the participants (caregivers of children living with CP) in their own settings. This was performed in order to understand the participants’ (caregivers’) perspectives and perceptions of the poorly understood phenomenon of execution of ADL and participation by their children in education, leisure and play activities.

### Research setting

Approximately 50% of the Limpopo province population lives in rural areas, and this province has the highest poverty level in SA (Maphumulo & Bhengu 2019). Data collection was conducted in the Modimolle–Mookgophong municipal area, in the Waterberg district. FH Odendaal is the hospital providing health services to areas in the Modimolle–Mookgophong municipality and is supported by only one health centre in the Mookgophong area and two clinics (in Vaalwater and Alma).

### Population and sampling strategy

The population for this study included all primary caregivers of children from 5 to 17 years with a confirmed diagnosis of CP who resided in Modimolle, Limpopo. A non-probability, purposive sampling frame was used to recruit the participants (Hunter et al. 2019). Participants were identified with the assistance of community care workers and a newly developed disability centre in Modimolle. The sample size was determined by identifying when data and meaning saturation had been reached (Aldiabat & Navenec 2018).

### Data collection

The interview schedule comprising questions that included probes was subjected to a peer-review process and piloted on caregivers of children living with CP in Vaalwater and Bela-Bela. The pilot study was conducted by the researcher over a period of one month (September 2019), and thereafter the interview schedule was adjusted accordingly. Data for the main study were collected over a two month period (October to November 2019). A data collection sheet (containing sociodemographic questions) and a semi-structured interview schedule (in-depth interviews) were used.

### Trustworthiness strategies

Credibility of data collection was optimised by using two audio-recorders during the interviews and all interviews were transcribed verbatim. Where participants responded in their vernacular, responses were translated by an independent Sepedi language expert and corroborated by the researcher. To ensure transferability, the researcher documented all environmental (physical and social) circumstances in which the data collection occurred. This was performed to enable the researcher in providing an accurate and rich description of the environment. Furthermore, to enhance confirmability, the researcher performed member-checking with the participants.

Member-checking ensures the credibility of the research data by providing participants an opportunity to confirm whether the data analysis and interpretations were a true reflection of their experiences (Speziale & Carpenter 2007). After data analysis, the researcher visited participants who consented to participation in the member-checking process to discuss the analysed data and interpretations. Caregivers who participated in the member-checking were satisfied with the data presented and the interpretation of their responses was a true reflection of their perceptions and experiences.

### Data analysis

In addition to the sample size recommended by Manson (2010), meaning saturation was assessed during the data coding process by considering the richness and thickness of data, which indicate data quality (Hennink, Kaiser & Weber 2019). Furthermore, operational saturation and theoretical saturation, as described by De Vos et al. (2011) and Forero et al. (2018) were achieved. Operational saturation is the process of assessing saturation from conceptualisation to finalisation of the study (Hennink et al. 2019; Hennink, Kaiser & Marconi 2017). Theoretical saturation is the point during category development at which no new categories emerge (Strauss & Corbin 1998). Saturation was achieved by quantifying the number of new codes per interview and mapping emerging data saturation patterns from the third participant (NVivo v11). Meaning saturation was observed after the 12th participant, however, analysis continued in order to achieve operational and theoretical saturation for all participants.

The bio-demographical quantitative data for the 19 caregivers and their children with CP are presented as frequencies, proportions and percentages. Central tendency is provided as median and interquartile ranges (IQR) (STATISTICA, www.statsoft.com) to describe the participant sample and provide the context to the qualitative enquiry. The transcribed audio files from the interviews were downloaded and anonymised by allocating a code to each respondent. The transcripts were imported into NVivo (version 11) for data management and further exploration of data (Kaefer, Roper & Sinha 2015).

The researcher analysed the data through the process of content analysis (Erlingsson & Brysiewicz 2017; Gaur & Kumar 2018). Coding was performed by the researcher and an expert colleague acted as co-coder. The coding process was initiated by identifying meaningful units through a process of condensation (Erlingsson & Brysiewicz 2017). Thereafter, manifest coding and latent coding were performed, and similar codes were grouped to form categories. Categories with similar trends were grouped into sub-themes. Sub-themes that were closely related were further grouped to form themes, and subsequently themes were combined to form overarching themes. When differences in coding and theme development arose, the two colleagues discussed in order to reach consensus. An overview of the themes, sub-themes, categories and participants’ quotes is provided in content analysis summary tables (Mathye & Eksteen 2016; Miles, Huberman & Saldaña 2014).

### Ethical considerations

Ethical clearance was obtained from the institutional ethics committee (SMUREC/H/94/2019:PG), and permission to conduct the study was granted by the Departments of Health, Social Development (Limpopo province) and the Chief Executive Officer (CEO) of the district hospital in Modimolle. Participants were also assured that participation was voluntary; all information was confidential and that they could withdraw from the study at any time without providing reasons or prejudice and health care would not be withheld from them. Written informed consent was obtained from the appropriate primary caregiver or guardian of the children concerned.

## Results

### Bio-demographic and socioeconomic data of caregivers and children

Twenty-two families were recruited, but three candidates were excluded as the age of the child had been incorrectly captured in the patient records. The final sample therefore consisted of 19 children and their primary caregivers (Table 1).

**TABLE 1:** Bio-demographic and socioeconomic data of caregivers and their children with cerebral palsy (*N* = 19).

Child (*N* = 19)			Caregiver (*N* = 19)		
Variable or category	Frequency	%	Variable or category	Frequency	%
**Sex**			**Sex**		
Male	1	5.26	Male	8	42.10
Female	18†	94.73†	Female	11†	57.89†
**Age (years)**			**Age (years)**		
20–29	3	15.78	5 – 8	4	21.05
30–39	4	21.05	9 – 12	8†	42.10†
40–49	7†	36.84†	13 – 17	7	36.84
50–59	3	15.78			
60–69	2	10.52			
**Number of dependants including the child with CP**			**Type of CP**		
0–1	5	26.31	Spastic diplegia	10†	52.63†
2–3	9†	47.36†	Athetoid	1	5.26
4–5	1	5.26	Hemiplegia	4	21.05
6–8	4	21.05	Spastic quadriplegia	3	15.78
			Hypotonia	5	5.26
**Level of education**			**Comorbidities or associated conditions**		
Primary school	3	15.78	Epilepsy	5	26.31
Secondary school (high school)	14†	73.68†	HIV Infection	2	10.52
Tertiary education	2	10.52	None	12†	63.15†
**Employment**			**Contractures**		
Not permanent	4	21.05	No contractures	8	42.10
Permanent	1	5.26	Single joint contractures	7†	36.84†
Self-employed	2	10.52	Multiple joint contractures	4†	21.05†
Unemployed	12†	63.15†			
**Marital status**			**GMFCS**		
Married	4	21.05	I	5†	26.31†
Not married	15†	78.94†	II	2	10.52
			III	3	15.78
			IV	5†	26.31†
			V	4	21.05
**Relationship with child**			**Mobility**		
Mother	14†	73.68†	Walking independently	6	31.57
Father	1	5.26	Walking frame	1	5.26
Grandmother	4	21.05	Buggy	2	10.52
			Wheelchair	9†	47.36†

CP, cerebral palsy; HIV, human immunodeficiency virus; GMFCS, Gross Motor Function Classification System.

† , highest number scored.

All caregivers who participated in this study were SA citizens; the majority were female and either the child’s parent or grandparent (21.05%). Most primary caregivers were unemployed, between 30 and 49 years old (57.89%) (median [IQR] age of 41.6 [31.3–49.8] years), and the majority were not married, either single or living together (78.94%). The highest level of education of most of the caregivers was secondary school (73.68%), with a reported median (IQR) of 3 (2–5) dependants.

The children living with CP who participated were mostly females (57.89%) and in the pre-adolescent or adolescent stage (median [IQR]: 11.0 [8.4–14.3] years). Most of the children were diagnosed with spastic CP in particular spastic diplegia (52.63%), presented with very few comorbidities and one or more contractures (57.89%). Based on the Gross Motor Function Classification System (GMFCS) level, the functional ability of most of the children was moderately to severely affected (median [IQR]: 3 [1–4]), and they used assistive devices such as wheelchairs (Table 1).

### Activities of daily living and participation patterns of children in education, leisure and play

The quantitative findings on participation patterns related to ADL, education, leisure and play of children with CP in Modimolle, as reported by their caregivers, are presented in Table 2.

**TABLE 2:** Quantitative data on children’s participation patterns in activities of daily living, education, leisure and play (*N* = 19).

Variable or category	Frequency	%
**Participation in ADL (self-care)** †		
Bathing and dressing (partially independent)	10	52.63
Feeding (partially independent)	15‡	78.9‡
Toileting (partially independent)	6	31.57
Fully dependant on caregiver for all ADL	4	21.05
Fully independent in all ADL	4	21.05
**Participation in ADL (family or community activities)** ‡		
Church	4	21.05
Household	5	26.31
None	11§	57.89§
**Participation in educational activities**		
Formal education	7§	36.84§
Informal education (for people living with disabilities)	6	31.57
None	6	31.57
**Participation in leisure and play activities**		
Play (other siblings, toys, alone, games)	11§	57.89§
Plays soccer	1	5.26
Watches television	3	15.78
Skipping rope	3	15.78
Swimming and boxing	1	5.26

ADL, activities of daily living.

† , Participants could at times perform more than one activity (e.g. feeding and toileting).

‡ , Participants could at times perform more than one activity (e.g. at home and at church).

§ , highest number scored.

As far as ADL, including self-care activities such as bathing and dressing, were concerned, the majority of the children were able to feed themselves (78.94%), while many could bath and dress themselves and were therefore only partially dependant on their caregivers (52.63%). Nevertheless, their caregivers still had to supervise or adjust the activity, for example, a child may have been able to bath and dress himself or herself, but set-up was required (e.g. the caregiver had to prepare the water and the clothes) and to stay close to assist whenever needed. The parents also reported that some of the children participated in community and family ADL such as religious (21.1%) and household activities (26.31%). In the case of children who did not participate in any activities (26.31%), their daily routine included bathing, eating, watching television or lying on the floor and sleeping. Four of the children were able to attend church services with their families on Sundays.

Approximately a third of the children with CP in Modimolle participated in formal education (36.84%), while 31.57% participated in informal education activities at the local centre for people with disabilities. The remainder of the participants (31.57%) were not participating in any educational programme at the time of the study. Over half the children were involved in activities, including play activities (57.89%) such as games on their phones and playing with toys. Others took part in active leisure activities such as skipping with a rope (15.78%) and soccer (5.26%) and passive leisure activities such as watching television (15.78%).

### Emerging themes

Four overarching themes emerged from the content analysis of the interviews: (1) bio-demographic and socioeconomic factors; (2) participation patterns in ADL, education, leisure and play activities; (3) barriers and (4) facilitators of participation. This article reports on the bio-demographic and socioeconomic context, as well as the participation patterns of children with CP living in the Modimolle–Mookgophong district.

### Performance of activities of daily living

Two sub-themes related to the performance of ADL emerged from the analysis of the data, namely: (1) assistance with ADL and (2) independence in ADL (Table 3).

**TABLE 3:** Participation in activities of daily living based on interviews with caregivers.

Theme	Sub-theme	Category	Direct caregiver quotes
Activities of daily living (ADL)	Assistance with ADL (partially independent or totally dependent)	Bathroom care (toilet and bath), self-grooming and feeding	‘Yes, I just bath him as though I am bathing a baby, but I put water in the dish [*basin*], I give him his washing rack and soap and I tell him to wash himself. He then stands up and cleans himself, once he is done, I then come in and bath him (sic).’ (P#015, mother of a boy with spastic diplegia) (cannot clean himself adequately).
			‘In the past, I would put the child on my lap. But seeing how the child has grown, I do not do that anymore, I use a spoon. The child would stick out the tongue. If I start feeding the child at 9 o’clock, I could finish at 10:30. That is when I can tell that the child is full. Sometimes when the child is full, you would see the head lean backwards, to show that the child is full.’ (P#016, mother of a girl with spastic quadriplegia) (takes a long time to feed the child).
		Home adjustments	‘So, she can have enough space in the bedroom because she finds it hard to get off the bed. Now she is unable to get on the couch.’ (P#017, mother of a girl with hypotonia) (caregiver is building a house with larger rooms so that the child will have enough space to move around).
			‘Yes, we made him a bath on the other side. There used to be a toilet here, I had it taken down. You could hear a person in the toilet. I had it taken apart and put in a bathtub and built a proper toilet and bathroom.’ (P#013, father of a boy with spastic quadriplegia) (family used small wash basins/bowls but had to build a bathtub for the child as he grew older).
		Household chores	‘Sometimes she would be on the bed trying to make the bed, hey, you would laugh. The pillows would be stretched out all over the place. Then I would make it straight.’ (P#017, mother of a girl with spastic quadriplegia) (trying to assist with household chores, although not always sufficient).
			‘Sometimes when I do laundry, I would take some of the items and put her on the chair, or on the floor, even on the couch. I sit on the other couch and tell her to fold them. She folds them well. When I chop morogo (spinach) I get her to join in. She will do it bit by bit.’ (P#017, mother of a girl with hypotonia) (trying to assist with household chores, but food preparation takes longer).
	Independence in ADL		‘Yes. Just cleaning. Like sweeping and mopping, vacuum, some of the dusting. I tried to teach her everything. Now, we are going to start learning cooking. She does not like it when her dad shows her, because he does not have patience, and everything must be done his way! And I would say, “Here is an onion, there is the potato, peel them, cut them.’ (P#021, mother of a girl with spastic diplegia) (can independently perform house chores).
			‘She can move around, and she is able to do her work, she interacts in every way.’ (P#019, grandmother of a girl with hemiplegia) (functionally independent).

The categories of assistance with ADL (children who are partially dependant) were:

- bathroom care (toilet and bath),
- self-grooming and feeding
- home adjustments
- household chores.

Although most children were able to participate in ADL and could assist with some household chores, caregivers explained that these children often needed assistance with some of the self-care ADL such as toileting, bathing and feeding. In the case of those who were fully dependant on caregivers for self-care and ADL, caregivers reported that they treated them like (little) children and did everything for them. Where children could perform some of these activities independently, their caregivers gave them the opportunity to do what they could and only assisted them with completing the task. Some caregivers even went as far as adjusting the home to enable their children to participate in some ADL and family activities.

Under the theme ‘independence in ADL’, a few caregivers reported that their children were fully independent when performing these activities and participated in household chores such as cooking, sweeping, and vacuuming. The sub-theme and categories are provided in Table 3, together with some verbatim caregiver’s responses.

### Participation in educational activities

Four sub-themes relating to participation in education emerged, namely: (1) participation in a formal education programme; (2) participation in informal education programmes; (3) no participation in any educational activities and (4) participation of other children with disabilities in the community.

As indicated in Table 4, children with CP in Modimolle participated in formal education activities but in a different way. The children in the early childhood programme (creche) were taught how to write, draw and even count, while children in mainstream education (‘attending school’) and those who were home-schooled were taught the national standardised curriculum. Those children attending public or private institutions as Learners with Special Education Needs (LSEN) were taught subjects such as English, Mathematics, isiZulu, as well as Arts and Culture, Physical Education, Computer Studies and Life Skills, and even ADL skills such as cooking and baking.

**TABLE 4:** Participation in educational activities based on interviews with caregivers.

Theme	Sub-theme	Category	Direct caregivers quotes
Educational activities	Formal education programme	Early Childhood Development (ECD)	‘Yes, now he attends crèche.’ (P#015, mother of a boy with spastic diplegia) (ECD).
			‘There were no problems at creche. The only issue is that the child used to go to… Creche here… for 2 years, then started attending school but I do not know if they taught them how to write, but they used to colour. Even if they coloured for them, I would not know because I was always at work.’ (P#019, grandmother of a girl with hemiplegia) (unsure whether the child’s learning is progressing).
		Mainstream education	‘It is the school I chose because the uncle, my last-born, attends there. Yes, he can grasp what they teach him. There are certain homework (sic) that he would come and show you, that he did at school. Others he would do here if there is something he is figuring out, or if he needs help, but you never have to do the work for him, he does it mostly by himself.’ (P#009, grandmother of a boy with spastic diplegia) (child is adjusting well to a mainstream school environment).
			‘After that the child started attending at … (primary school). The child started well in Grade R, then Grade 1 and passed well, then did Grade 2 and passed. I was not that impressed with the marks in Grade 2, and I noticed that reading seemed challenging, so I said the child must repeat. The child should be in Grade 5, but I insisted the child repeat because I was not satisfied with the progress. The child went back and repeated Grade 2 and passed and went to Grade 3… then passed Grade 3 and went to Grade 4. Now I do not know how the child reached this stage only to struggle in Grade 4. But there is some effort to do better.’ (P#019, grandmother of a girl with hemiplegia) (learning progress is slower than typically developing peers).
		Special school education (school for children with special educational needs [LSEN])	‘I found him a school this year, at …. It is a school for children with disability or slow learners. They teach them language, they teach them writing, they also teach them handy crafts (sic).’ (P#020, mother of a boy with hemiplegia) (it is difficult to find a special school for children with disabilities who live in Modimolle).
			‘Yes, they assess the child’s strengths and start with the subjects, handiwork, baking…. Yes, she would ask me that we get ingredients from the store and that she will show me what to buy.’ (P#022, Mother of a girl with hemiplegia) (child enjoys the curriculum and/or activities provided at school).
	Informal education programme	Participation in educational activities at the local disability centre	‘The only one I know, I am not sure if you are aware of it, down at the disability centre. It is usually [for] children who can hold things … their disability is not severe. Others in wheelchairs, they go there and get meals and that’s it. The other time, we attended an event there, seeing donations being made and so on. They hosted them to a lovely meal. Some can speak and so on.’ (P#018, mother of a girl with spastic quadriplegia) (a well-known place where children with disabilities participate in activities).
			‘I think she is safe there [disability centre]. Because when I ask her what she learned at school, she always tells me about prayer. She can pray the Lord’s prayer.’ (P008, grandmother of a girl with hemiplegia) (learning progress observed).
	No participation	Does not attend school or centre	‘P#005 is getting older and there was mention of a school for children with disability. She goes there (to hospital) for treatment and we were told that they would start going to school when the time comes and because they go for treatment, their fees should be paid by the hospital. As they promised to speak to a social worker to then come and explain what needs to happen going forward, and I am still waiting.’ (P#005, mother of a boy with spastic quadriplegia) (not knowledgeable about the process of enrolling their child in a school for children with special needs).
			‘Heard that she was starting a school and I went to register the child [disability centre], spoke with his parents and registered him, and he went there. The parents would assist with school fees, but later, they said they did not see any progress from the school. They said the lady was not teaching them anything, and it was the same as if he was staying at home – they could not see any progress.’ (P#010, grandmother of a boy with spastic quadriplegia) (no learning progress observed).
	Participation of other children with disabilities (in the community of Modimolle)	Attends a special school	‘Yes. I know children like that, I know children who have body disability, I know children with brain damage. Children like that … they all go to … [special school].’ (P#021, mother of a girl with spastic diplegia) (only knows of children with disabilities that go to a special school).
			‘The ones I know are older, they go to … [special school]. That is the school they attend ….’ (P#002, mother of a girl with spastic quadriplegia) (only knows of older children with disabilities who attend school).
		School or academic progress	‘I do not know how far they go. Yes, I only see them going to school. We would come across them and I would say that if my child could walk like them, she would also be going to school.’ (P#005, mother of a girl with hypotonia) (school or academic progress unknown).
			‘I only see them going to school. I do not know of anyone that completed there, and what they are up to now.’ (P#002, mother of a girl with spastic quadriplegia) (academic progress of children with disabilities unknown).

LSEN, Learners with Special Education Needs; ECD, Early Childhood Development.

Under the sub-theme ‘informal education’ (Table 4), children participated in educational activities at the local disability centre. These children were taught to pray, to write and to participate in physical education activities such as wheelchair races. The category of ‘no participation in educational activities’ included caregivers who reported that their children stayed at home and did not attend any school or centre.

The sub-theme ‘other children living with disabilities’ described caregivers who observed that children in their community attended special schools; however, they could not recall any of these children participating in educational activities beyond basic school education (no tertiary education). Other caregivers had seen children with disabilities who were ambulant undergo basic education at a school or centre and not necessarily those who were unable to walk.

### Participation in leisure and play activities

Four sub-themes related to participation in leisure and play activities emerged, namely (1) leisure and recreational activities (at school, home or in the community); (2) social or religious gatherings; (3) loss of interest in participation as child grows older and (4) programmes for typically developing peers (Table 5).

**TABLE 5:** Participation in leisure and play activities based on interviews with caregivers.

Theme	Sub-theme	Category	Quotes
Leisure and play activities	Leisure or recreational activities (at school, home or in the community)	Sport (e.g. football or soccer)	‘They have fields at the back there. They would play football after school or on weekends.’ (P#009, grandmother of a boy with spastic diplegia) (play with friends).
			‘Yes, she had been running at school during sports competitions. She used to also play netball. She cannot hold the netball well because of her hand. She loves these things but I am sure they realise that she cannot go far. I also have not been to the school to ask about her running, I have seen her running here [at home]. She runs very differently.’ (P#019, grandmother of a girl with hemiplegia) (motivated and wants to participate in a variety of sports).
		Playing in the street; dance steps	‘They just play around. They would be jumping there around here nearby.’ (P#008, grandmother of a girl with hemiplegia) (the children play near home).
			‘There is a place where others go and dance steps.’ (P#022, mother of a girl with hemiplegia) (traditional wedding dance).
		Watching television	‘As she can’t always stay in the bedroom, we bring her to watch some TV. When she watches TV, I have to sit in a way that she can lean on me. And I am the only one who can manage her. I sometimes prepare for her to lay down outside. That is the only way – it is either she lies down outside and gets some fresh air, or we sit here.’ (P#018, mother of a girl with spastic quadriplegia) (child requires full-time supervision or support).
			‘We would be sitting there, watching TV, and she would be leaning against me.’ (P#017, mother of a girl with hypotonia) (mother-daughter bonding).
		Physical education	‘It looks as if they (staff at the disability centre) want to teach them how to play tennis with wheelchairs, like we see on TV.’ (P#007, mother of a girl with spastic quadriplegia) (local disability centre encourages physical activity and participation in sport).
			‘Unless at the school. That is where they do activities. When they have finished their work in the classroom, they let them out. They could let them sing or take those balls and exercise with them. There are various activities.’ (P#017, mother of a girl with hypotonia) (school encourages physical activity and participation in leisure and play activities).
		Spectators	‘His uncle, my last born, who goes to college, takes him to play when he is back from college. He likes basketball, so he would go with him all over …. It is with ordinary children; they play ball together. He cannot stand so he would only watch and enjoy.’ (P#010, grandmother of a boy with spastic quadriplegia) (family supports/encourages the child to participate or be outside and watch others).
			‘They would play with cars, and the child would watch and laugh.’ (P#016, mother of a girl with spastic quadriplegia) (passive participation).
	Social or religious gatherings	Attending church services or functions (e.g. weddings)	‘She even carries children at the church. We would be nervous that she might drop them, but it never happens.’ (P#019, grandmother of a girl with hemiplegia) (interactive with other children at religious gatherings).
			‘If there are weddings or parties, I take her and put her on the chair. Later, we’d come back.’ (P#017, mother of a girl with hypotonia) (social/community participation and interaction).
		Visiting neighbours or community or family members	‘I will take him along if I want to go and visit somebody around, like visiting one of my lady friends, I will go with him.’ (P#010, grandmother of a boy with spastic quadriplegia) (visits caregiver’s friends).
			‘He spends a lot of his time with his family. We take him to visit family.’ (P#013, father of a boy with spastic quadriplegia) (spends time and interacts with extended family).
	Loss of interest as they grow older		‘She would go to them that side. But now she does not want to go there. She would have to cross to the other side, and it is at a distance. The lady there passed away, that is where she liked to go. She would go to another house that other side. There are lots of people there. I do not know why she decided not to go anymore. She just stopped on her own. I remember asking her if she is not going to visit them, and she said that they would have to come to her. I do take her sometimes.’ (P#017, mother of a girl with hypotonia) (physical ability influences participation patterns and social interaction).
			‘A lot of the children come and play with him here, but he cannot go to where they stay, even if you open the gate, he will not go out across there.’ (P#015, mother of a boy with spastic diplegia) (inability to travel far distances, fear of moving outside the home environment).
	Programmes for typically developing peers	Sports: boxing, running and soccer	‘They can go and do boxing and, you know, karate is there, there is rugby, there is cricket, there is hockey, gymnastics, normal stuff for children. But for mine, no.’ (P#021, mother of a girl with spastic diplegia) (inequality in sport/development programmes and opportunities for children with disabilities and for their typically developing peers).
			‘Down there, they usually play soccer. But I only see grown-ups and not young ones like [*my son*]. If there were children of his age, he would play with them.’ (P#001, mother of a boy with spastic diplegia) (only older children participate in the community leisure activities).

In the category ‘leisure and recreational activities’ (at school, home or in the community), caregivers reported that their children participated in recreational activities such as sports (e.g. football and netball), playing alone or with toys, while some even played in the street with other children. The children also participated in recreational activities, such as doing dance steps and skipping with a rope with friends and family; however, because of their physical difficulties with jumping most children preferred to be the one holding the rope rather than the one jumping. Those who were more ambulant also participated in swimming and boxing in their parents’ yards, while others had the opportunity to participate in physical education at the disability centre in their area. Those who were unable to participate physically in play activities would either watch television or watch other children playing sports or games in their leisure time.

In the category ‘social or religious gatherings’, caregivers reported taking their children to functions such as weddings and church services and visiting neighbours or family members. In the category ‘loss of interest in participation with age’, caregivers raised the concern that their children tended to lose interest in playing, especially outside their homes, as they grew older and also because of regression in physical function and fear of being teased.

In the category ‘programmes for typically developing peers’, caregivers reported that typically developing children were exposed to programmes such as boxing, karate and cricket in the community and therefore were able to participate in more activities than their peers with CP.

## Discussion

### Bio-demographic and socioeconomic context of participants

The age and marital status profile of caregivers in this study are similar to that of a study conducted in Gauteng among caregivers of children living with disabilities (Kropiwnick, Elphick & Elphick 2014). In this descriptive study in Modimolle, the highest level of education of caregivers was, in most cases, secondary school. This aligns with STATSSA (2011), which reported that 81.2% of black SA citizens over the age of 20 years had completed primary and secondary education only (https://www.statssa.gov.za).

Similarly, only two of the caregiver participants in this study reported having a tertiary qualification, which could explain the high unemployment rate among this cohort of participants. Eighteen of the 19 participants lived in an informal settlement and experience inaccessibility of services and scarcity of resources. This result agrees with the studies by Christian (2014) and Maphumulo and Bhengu (2019), which highlights the difficulty in accessing services and the limited resources experienced by children living with disabilities in SA.

The Limpopo province is among those SA provinces where 58% of citizens receive social grants as their primary source of family income (Statistics South Africa [STATSSA] 2015). A survey by STATSSA (2011) showed that 47.6% of the households in Limpopo province depended on social grants as their main source of income, with three to five social grants per household and a median (IQR) of 3 (2–5) dependants per caregiver. These results are similar to those reported by Kelly (2019) in a study on disability, cash transfer and family practices in SA, where their findings indicated the sharing of social grants among families.

In this study, there were more female than male children; these findings differ from those of the study by Kropiwnick et al. (2014), who found that the majority of children with CP in Gauteng who participated in their study were male. A possible reason could be the difference in sex distribution between the Limpopo and Gauteng provinces. The male-to-female ratio in the Gauteng province is different from that of Limpopo, as Limpopo has more females (Statistics South Africa [Stats SA] 2019).

The study by Kropiwnick et al. (2014) included children from a broader age range (including younger than 5 and older than 18 years). Furthermore, similar to the present study, the majority of the children in the study by Kropiwnick et al. (2014) had been diagnosed with spastic diplegia with resulting contractures, decreased range of motion in numerous joints, fatigue and pain, a commonly expected clinical picture of children with CP (Bangash et al. 2021; Donald et al. 2014; Mutlu, Bugusan & Kara 2017; Reddy 2005; Richards & Malouin 2013). The comorbidities epilepsy and HIV were found in this study’s patient cohort, as in other studies in Africa (Donald et al. 2014; Eunson 2012, 2016; Odding et al. 2006).

As for the level of mobility and ambulation of the children in this study, there was a wide distribution, as indicated on the GMFCS scores. Just under half the participants presented with a GMFCS score of IV or V, indicating that these participants were permanent wheelchair users. This fact is supported by the findings of Palisano et al. (2010) in Ontario (Canada), which indicated that the probability of wheelchair usage in children classified as GMFCS IV increases with age.

### Participation in activities of daily living, education, leisure and play

Participation in an array of activities assists in learning life skills such as social skills, problem solving and self-identity awareness, which may facilitate children’s potential to experience social fulfilment, intellectual achievement and to live an economically productive life (Aron & Loprest 2012; Bourke-Taylor et al. 2017; Majnemer et al. 2010; Orlin et al. 2010; Shikako-Thomas et al. 2013). Participation, therefore, contributes to children reaching their highest possible level of independence (Aron & Loprest 2012).

Although most children in this study were able to perform ADL, they needed assistance with some aspects, such as self-grooming, toileting, bathing and feeding (Table 2). This could be explained by the fact many of them presented with a functional mobility level (GMFCS) of III–V, implying that their physical disability ranged from moderate to severe (Table 1). According to McManus, Corcoran and Perry (2008), children with GMFCS scores of III–V are dependant on caregivers for their ADL. In a study by Amaral et al. (2014), it was found that despite their disability, children with CP participated in certain household chores. These findings correlate with those of this study that children were participating in some household chores although they were dependant on caregivers for some of their ADL. In this study in Modimolle, a few caregivers had made home adjustments to assist their children to become more independent. Some caregivers were unable to adjust their homes, for instance, to add ramps, railings and bathrooms because of a lack of financial resources brought about by both the financial burden of taking care of a child with CP and unemployment (Kelly 2019).

Although all the participants in this study were of compulsory school-going age (5–17 years) and despite the documented advantages of participation in educational activities for children with disabilities (Aron & Loprest 2012; Bourke-Taylor et al. 2017; Shikako-Thomas et al. 2013), only two thirds of the sample participated in formal and informal education. The school attendance rate in this study was much lower than the study by Nuga-Deliwe (2016), where it was found that 83.3% of children with disabilities in SA were attending mainstream schools or schools for learners with special needs. Although similar educational services are available to children in the Modimolle–Mookgopong district, full-service school programmes for children with disabilities as prescribed in Education White Paper Six (EWP6) are not available (Department of Education 2001). Reasons for the lower rate of school attendance in Limpopo could be that it is one of the poorest and most underdeveloped provinces in SA with limited services for people with disabilities such as schools and hospitals (Maphumulo & Bhengu 2019; STATSSA 2016; STATSSA.gov.za 2019).

In this study, caregivers responded that the children in their care attended early childhood education programmes, a mainstream school, a local disability centre or a school for children with special needs. A study by Morse and Bell (2018) found that caregivers of children with disabilities opted to home-school their children, as they felt that their educational needs were not being met within the educational structures in the community. Similarly, in this study, the one caregiver who chose to home-school her child reported that her child was not provided with enough support in the mainstream school. The low school attendance rate among these children corresponds to the results reported by the Department of Basic Education (DBE 2015), which indicated that 43.85% of children with disabilities did not attend school (Nuga-Deliwe 2016). The DBE survey, however, focused only on children in public schools and did not include those attending private institutions (Nuga-Deliwe 2016). The results of this study indicated that 30% of children were educated at a disability centre, which does not fall under the jurisdiction of the DBE.

In the case of children living with disabilities in the Modimolle–Mookgophong district who were not part of the study, the caregivers in this study reported having seen these children attending schools for children with special needs. However, the caregivers did not know whether any of these children had received education beyond their basic education. Ndlovu and Walton (2016) reported that children with disabilities encountered more difficulties with access to educational institutions. They also experience limited support from disability units at tertiary institutions, because of unimplemented policies, inadequate funding and inaccessible buildings, public transport, the environment and negative attitudes in the community. These aspects of post-basic education for children with CP require further exploration.

As far as leisure activities are concerned, children who were reported to be more ambulant (Table 2) participated in a variety of recreational activities such as football or playing on their own or with their toys or with other children in the street. Other recreational activities included doing dance steps, skipping rope, sports and physical education activities at the disability centre. Some children were fortunate to participate in swimming and boxing, while others preferred social activities including joining the caregiver during social gatherings, such as church, weddings and even visiting neighbours. These types of activities were similar to the recommended recreational and social activities identified by the Children’s Assessment of Participation and Enjoyment (CAPE) outcome measure (King et al. 2004) and leisure activities reported among children with CP living in Spain and the Netherlands (Bult et al. 2011).

Caregivers in Modimolle reported that children who were not able to actively participate, participated passively by simply watching others play, either in the streets or on the fields where games such as basketball were played (Table 3). This supports the findings of Alghamdi et al. (2017) regarding the negative impact of physical impairment on children’s participation in recreational activities. Those children who could participate in activities such as boxing, cricket and running in the community were, according to their caregivers, those who presented with less severe physical impairments. This corresponds to the results of a study by Majnemer et al. (2008) who found that children living with disabilities were involved in fewer activities than their typically developing peers.

### Strengths and limitations

This study provides insights into the participation patterns of children with CP living in a rural setting in SA. The methodology was designed to gather information to explore the human experience from the perspective of caregivers of children living with disabilities. Eighteen of the participants lived in an informal settlement in a rural area that could have skewed the data and limits the generalisability of the findings. Accessibility of public facilities such as education and entertainment facilities or schools and parks in the Modimolle–Mookgophong district were not assessed which constitutes a limitation.

### Implications and recommendations for practice, research, and policy development

Children living with CP in the Modimolle–Mookgophong district participated in certain activities within their communities. However, further investigation into the barriers and facilitators influencing their participation patterns and the academic performance of children living with disabilities in rural areas such as Modimolle is required. Based on the findings of this study, the researchers recommend that local government (the municipality), the Departments of Basic Education, Social Development and Health should collaborate to create and optimise appropriate educational and recreational (leisure and play) opportunities for children living with disabilities. Development and implementation of community-specific, integrative health and social care strategies to enhance participation among children living with disabilities are recommended.

Furthermore, legislative support and policy implementation are required from the South African government to improve participation and integration of children living with CP. Caregivers should also be supported in accessing treatment options for improving children’s independence in participation and in making appropriate adjustments to their homes to enhance their children’s independence when engaging in ADL.

## Conclusion

This study was performed in an informal settlement and rural area of SA where families have limited access to services and resources. Although children with CP residing in Modimolle perform some ADL and participate in educational, leisure and play activities, they are not fully integrated into their community. Furthermore, the rate of participation in activities was low and less diverse than that of able-bodied children and participants’ interest declined with age. Legislative support and policy implementation are required to improve the participation and integration of children living with CP. Further studies on community-specific integrative strategies to enhance participation among children living with disabilities are recommended.
